# Thermal and Chemical Characterization of Digital Light Processing (DLP)-Manufactured Polymer Composites Reinforced with Jute Fibers

**DOI:** 10.3390/polym17111504

**Published:** 2025-05-28

**Authors:** Raí Felipe Pereira Junio, José Carlos Ferreira Fontes, Douglas Santos Silva, Bernardo Soares Avila de Cêa, Sergio Neves Monteiro, Lucio Fabio Cassiano Nascimento

**Affiliations:** 1 Military Institute of Engineering—IME, Department of Materials Science, Praça General Tibúrcio, 80, Praia Vermelha, Urca, Rio de Janeiro CEP 22290-270, RJ, Brazil; raivsjfelipe@ime.eb.br (R.F.P.J.); bernardo.soares@ime.eb.br (B.S.A.d.C.); sergio.neves@ime.eb.br (S.N.M.); lucio@ime.eb.br (L.F.C.N.); 2 Department of Civil Engineering, Augusto Motta University Center—UNISUAM, Avenida Paris, 84, Bonsucesso, Rio de Janeiro CEP 21041-020, RJ, Brazil; jc.ferreirafontes@hotmail.com

**Keywords:** additive manufacturing, digital light processing (DLP), jute fibers, thermal properties, chemical properties

## Abstract

The growing demand for sustainable materials with tunable thermal and structural properties has driven the development of composites reinforced with natural fibers in additive manufacturing (AM) technologies. This study investigates the thermal and chemical behavior of polymer composites produced via Digital Light Processing (DLP), an AM technique based on vat photopolymerization that stands out for its high resolution, dimensional control, and superior speed compared to methods such as FDM and SLA. Samples were manufactured with a UV-curable acrylate resin reinforced with jute fibers (*Corchorus capsularis*) in mass fractions of 0%, 2%, 2.5%, and 3% in solid geometries (CS-). TGA indicated a 4% reduction in the initial degradation temperature with increasing fiber content, from 326.3 °C (CS-0) to 313.2 °C (CS-3.0). TMA revealed a reduction of up to 19% in the coefficients of thermal expansion, indicating greater dimensional stability. The DMA indicated a 16.9% decrease in the storage modulus with 3% fibers, evidencing changes in the viscoelastic response. FTIR detected additional bands at 3340 cm^−1^ and 1030 cm^−1^, related to O–H and polysaccharides, confirming a fiber–matrix chemical interaction. These results demonstrate the potential of jute as a sustainable reinforcement in photopolymerizable resins, paving the way for ecological and functional applications in 3D-printed composites.

## 1. Introduction

The growing demand for sustainable and high-performance materials has significantly driven the advancement of polymer composites reinforced with natural fibers, aligning with global efforts to mitigate environmental impact and promote the principles of a circular economy [1,2,3,4,5,6,7,8,9,10]. Among these, lignocellulosic fibers such as jute (*Corchorus capsularis*) have emerged as promising alternatives to synthetic fibers due to their biodegradability, renewability, low cost, and favorable specific mechanical properties. These attributes have rendered them suitable for applications in the automotive, aerospace, packaging, and construction industries [11].

In parallel, additive manufacturing (AM) technologies, particularly Digital Light Processing (DLP), have revolutionized the production of complex components, offering significant advantages such as geometric freedom, mass customization, and efficient material utilization. DLP is a vat photopolymerization technique that uses ultraviolet (UV) light to selectively cure photopolymer resins, enabling high-resolution fabrication with excellent dimensional accuracy. Compared to conventional manufacturing methods, DLP facilitates the rapid prototyping of intricate geometries with controlled microstructural features [12,13].

The integration of natural fibers into photopolymer matrices via DLP presents a compelling opportunity for developing sustainable composite materials. However, several challenges must be addressed, including the compatibility between the hydrophilic nature of natural fibers and the typically hydrophobic resin matrix, the uniform dispersion of fibers within the matrix, and the influence of fiber incorporation on the thermal, chemical, and mechanical properties of the final composite. Although substantial progress has been made in incorporating natural fibers into thermoplastics via Fused Deposition Modeling (FDM), studies focusing on their application in UV-curable systems using DLP remain limited [14,15,16,17,18].

In addition to the growing attention given to lignocellulosic fibers as sustainable alternatives to synthetic reinforcements, it is also essential to discuss the sustainability of the polymer matrix used. Photopolymerizable resins used in additive manufacturing processes, such as Digital Light Processing (DLP), are traditionally derived from petrochemical sources and, once cured, become thermoset materials with cross-linked three-dimensional structures. This irreversible cross-linking, although providing excellent mechanical properties and dimensional stability, significantly limits the possibility of recycling by conventional methods.

However, recent advances have explored the chemical modification of these resins to make them reprocessable or partially biodegradable, through the introduction of dynamic units or bio-based monomers. For example, researchers have developed photopolymerizable resins based on vanillin and dimerizing diamine grease, containing dynamic imine bonds, which allow thermal reprocessability of the cured materials [19]. Furthermore, initiatives such as the VitriMore project are focused on the development of recyclable biobased resins for 3D printing, using vitrimers that combine the stability of thermosets with the recyclability of thermoplastics [20].

Digital Light Processing (DLP) offers distinct advantages in the fabrication of fiber-reinforced photopolymer composites. It employs a photoreactive system typically composed of acrylate monomers (reactive diluents), oligomers (prepolymers), and photo-initiators. The acrylate monomers determine key factors such as viscosity, the cure rate, polymerization shrinkage, and post-cure mechanical properties. Oligomers form the structural backbone of the cured resin, contributing to its toughness, environmental stability, and optical clarity. Photo-initiators generate free radicals under UV exposure, initiating the polymerization process. The control over these components is critical for tailoring the performance of DLP-fabricated composites.

Despite its advantages, the literature reveals a knowledge gap concerning the use of lignocellulosic fibers in DLP-based systems. The majority of current studies remain confined to traditional thermoset and thermoplastic matrices processed through extrusion-based AM techniques. This underlines the need for research exploring how natural fibers interact with photopolymerizable matrices, particularly with respect to their thermal decomposition, dimensional stability under temperature variation, and interfacial bonding mechanisms.

The thermal stability of polymer composites is intrinsically linked to their molecular structure and morphology. The presence of natural fibers, such as jute, significantly influences the thermal properties of the material, due to its lignocellulosic nature that can accelerate thermal degradation. Studies have shown that the interaction between the polymer matrix and fibrous reinforcements directly affects the thermal stability of composites. For example, the addition of nanocellulose can improve thermal stability due to the strong interfacial interaction between the matrix and fibers [21,22,23,24,25,26,27].

Furthermore, the semicrystalline structure of the polymer matrix plays a crucial role in thermal stability. Materials with a higher degree of crystallinity tend to have better thermal resistance, as the crystalline regions act as barriers to thermal propagation. Thermogravimetric analysis (TGA) is an essential tool for assessing the thermal stability of composites, allowing the identification of degradation temperatures and decomposition mechanisms. This information is essential for the development of materials with optimized thermal performance.

Thus, this study addresses this gap by investigating the thermal and chemical behavior of polymer composites produced via DLP using an acrylated resin matrix reinforced with short, untreated jute fibers. Composites were fabricated with varying fiber mass fractions (0%, 2%, 2.5%, and 3%) and characterized using a combination of thermogravimetric analysis (TGA), Differential scanning calorimetry (DSC), thermomechanical analysis (TMA), and dynamic mechanical analysis (DMA), along with Fourier transform infrared spectroscopy (FTIR). This comprehensive analytical approach provides valuable insights into the structure–property relationships of the developed biocomposites, demonstrating how natural fiber reinforcement can be leveraged to tune the thermal and mechanical performance of DLP-fabricated materials. The findings contribute to the advancement of eco-friendly and high-performance materials for emerging engineering applications in additive manufacturing.

## 2. Materials and Methods

### 2.1. Light-Curing Resin

**Figure 1:**
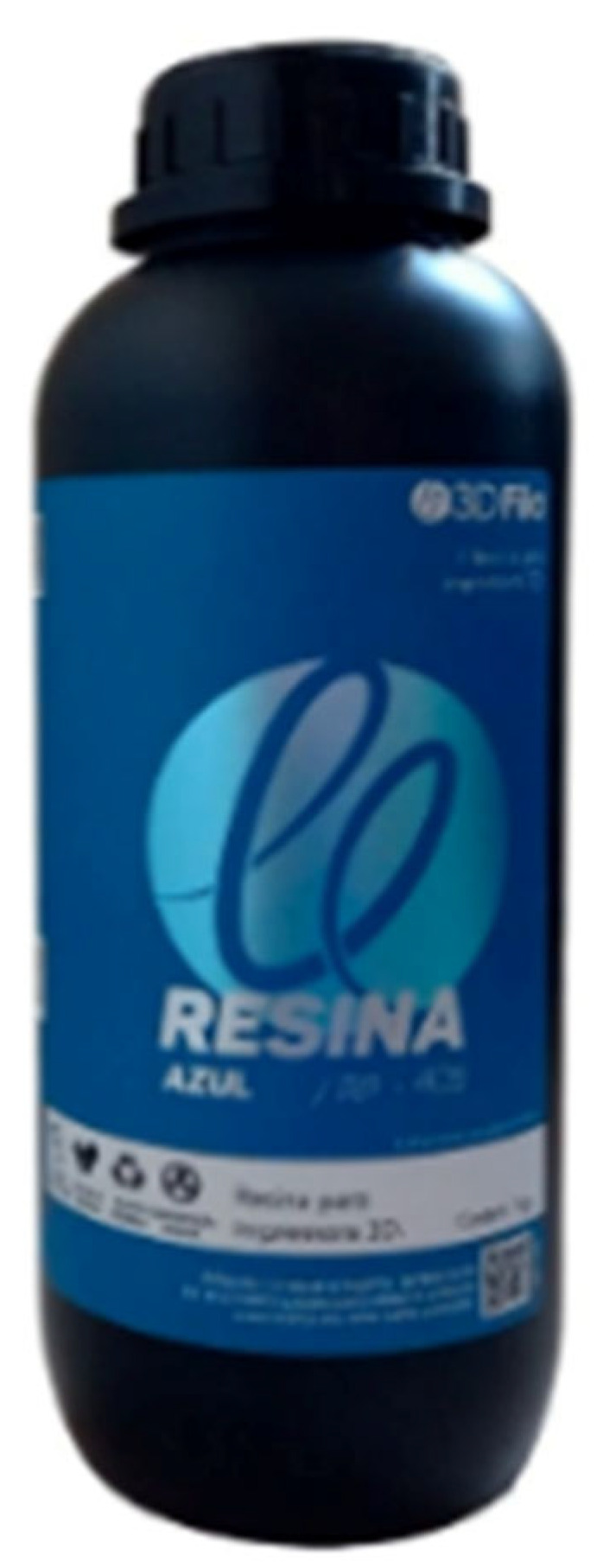
Photopolymerizable resin utilized for printing samples with DLP (3D Fila Azul Matte).

To create the samples via additive manufacturing, 3D Opaque Blue resin (405 nm) (Figure 1) was utilized, produced, and offered by D3M INDUSTRIA E COMERCIO LTDA. The polymer was created for printers utilizing SLA, DLP, or LCD Digital Light Processing technology. It is a substance known for its rapid polymerization rate, quick curing process, and excellent final print quality.

The manufacturer’s technical details regarding the polymer utilized are displayed in Table 1.

### 2.2. Jute Fibers (Corchorus Capsularis)

In this study, jute fibers (*Corchorus capsularis*) were used as additives to the light-cured resin for the manufacture of composites by DLP. The fibers were in the form of fabrics with an intermediate weave of T4, from the distributor of natural fiber fabrics, Sisalsul Ltda.

**Figure 2:**
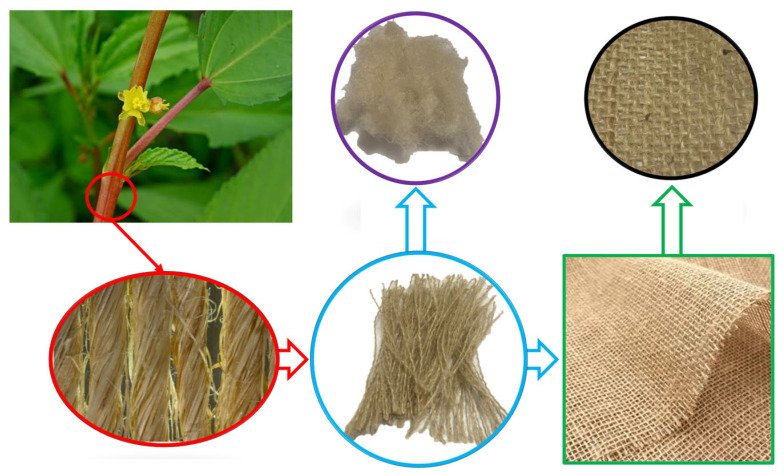
Cloth, lengthy strands, and crushed jute fibers.

The jute fabrics purchased were not suitable as additives in the DLP additive manufacturing process, since the limitations of the process prevent the use of large fabrics or fibers. To prepare the fabrics for the process, they were defibrated and processed in a knife mill for grinding. After grinding, the fibers underwent a particle size separation to ensure the dimensional regulation of the fibers used in the additive manufacturing process. Sieves with openings of 1.40; 0.71; 0.5; 0.18; 0.125; 0.09, and 0.063 mm were used. Figure 2 shows the fabric cutouts along with the fibers that were removed and ground.

After the screening process, the fibers that passed through the 0.18 mm sieve were selected for the production of composites.

### 2.3. Manufacturing of Composites

The composites were developed via DLP by the fiber mass fraction contained within them. Therefore, composites containing 2.0%, 2.5%, and 3.0% mass fraction of jute fibers were produced. The photopolymerizable resin composites reinforced with jute fibers were developed using the additive manufacturing technique via Digital Light Processing (DLP). Initially, the jute fibers were subjected to a cleaning process with running water to remove surface impurities, followed by drying in an oven at 60 °C for 24 h, with the aim of eliminating residual moisture, a critical factor to ensure good fiber–matrix adhesion and avoid defects during printing. After drying, the fibers were cut using a knife mill, ensuring a reduction in their length to values compatible with the process. Subsequently, a dimensional selection was performed by sieving, aiming to obtain a more homogeneous size distribution, favoring uniform dispersion in the polymer matrix. The fiber incorporation step in the photopolymerizable resin occurred by means of a mechanical mixer, operated for 10 min, ensuring adequate mixing and the breaking of any agglomerates. The mixture obtained was carefully placed in the DLP printer tank, where the specimens were printed, according to the previously established parameters for geometry and printing orientation. After printing, the pieces were subjected to a cleaning process with isopropyl alcohol to remove residues of unpolymerized resin, followed by a post-curing step in an ultraviolet light chamber (405 nm) for 24 h, ensuring complete cross-linking of the polymer matrix and dimensional stabilization of the samples. Table 2 displays the proportions assigned to each condition during the production of the composites.

A standard printing volume of 125 mL of resin was selected for the composites, as the printer requires a minimum resin volume of 100 mL for operation. Consequently, the designated value meets the minimum resin volume requirement for proper equipment function.

The samples were labeled based on the fiber mass fraction in the composite. Table 3 presents the naming conventions used for each composite condition created in this study.

Due to the cutting and screening of the jute fibers, they possess a low aspect ratio, which means that the samples produced through additive manufacturing are categorized as polymer matrix composites featuring discontinuous and randomly arranged fibers [28].

### 2.4. Approach Utilized for Additive Manufacturing

To ensure the proper application of additive manufacturing, several procedures were executed until the components were produced. The process of utilizing AM in this research can be outlined as follows:

- Creation of the digital model;
- Transformation into the “.stl” format;
- Cutting;
- Fixing issues with design, slicing, and printing;
- Post-treatment of the produced components.

#### 2.4.1. Development of the Digital Model

The creation of the digital model was founded on the geometric specifications mandated by the standards used for the characterizations applied to the samples throughout this study’s development. Every digital representation of the samples created in this research was developed using AutoCAD 360—AutoDesk^®^ (Educational License) CAD software.

#### 2.4.2. Change to “.stl” Format

**Figure 3:**
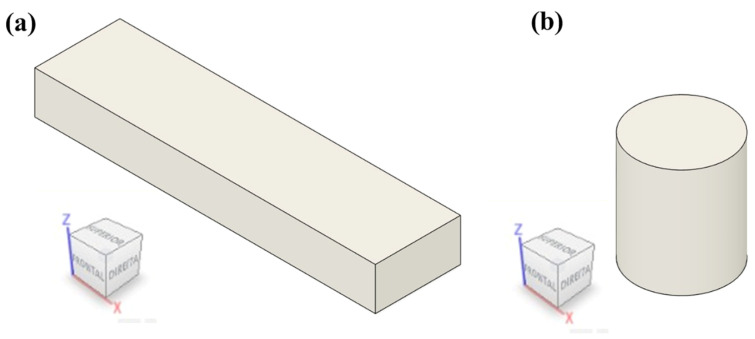
Digital models transformed using Fusion 360^®^ software: (**a**) DMA test samples and (**b**) TMA test samples.

The digital model created with CAD software was transformed into “.stl” format. This format is referred to as the standard tessellation language, which retains details regarding the geometry of the model’s surface. The digital models were converted to the “.stl” format using Fusion 360—AutoDesk^®^ software (Educational license). Figure 3 illustrates several models transformed into the “.stl” format in the current research.

#### 2.4.3. Cutting

**Figure 4:**
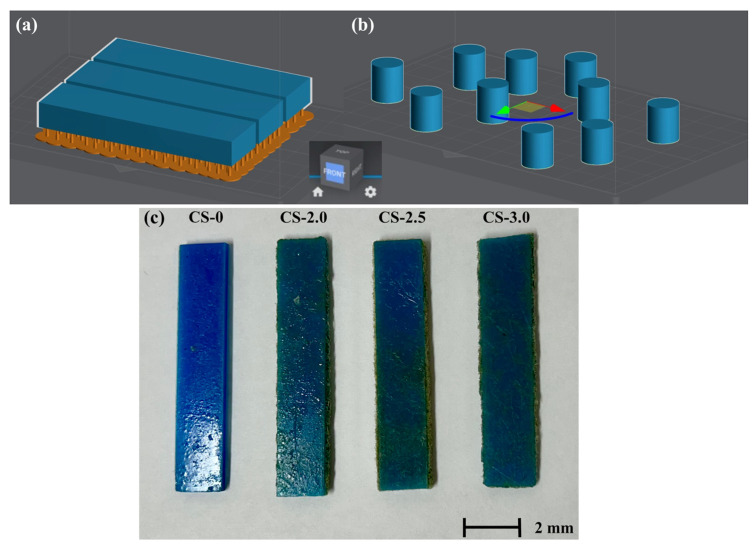
Utilizing Lychee Slicer^®^ Software to cut the sample designs: (**a**) DMA test samples, (**b**) TMA test samples, and (**c**) DMA samples developed.

Once converted, the “.stl” file needs the model to be sliced, and, for this purpose, the LycheeSlicer^®^ software was utilized. Slicing is essential for assessing the quality of printed components, as the software employed produces printing codes from the data in the “.stl” file. The generated code resembles CNC machine codes that outline the platform’s movement while printing. Figure 4 demonstrates how LycheeSilicer^®^ was utilized to create the printing code for various samples.

#### 2.4.4. Error Correction in Slicing

Following the slicing procedure, it is typical to notice design mistakes. To address these issues, the UVTools^®^ software was utilized. The software analyzes every layer produced in the “.stl” file pixel by pixel, detecting potential printing mistakes. This scan enables adjustments and prevents issues that might arise throughout the procedure.

#### 2.4.5. Printing

Once the digital model was created, converted, sliced, and printing errors corrected, the resulting model was prepared for the printer, allowing the component to be produced as intended. To achieve this, the Creality Halot One CL-60 3D printer was utilized. To produce the engineered components, the machinery needed to be set up for printing, utilizing configurations that enabled the printing of pure resin components and composites. The parameters used for creating the pure resin test specimens were applied as specified by the manufacturer of the equipment and resin. For the composite production, parameters were chosen that would enable the creation of components with satisfactory quality. Therefore, the equipment setups used for the resin and composite components are shown in Table 4.

The Creality Halot One CL-60 printer features a bottom-up design, 405 nm LED light source, offering a printing volume of 127 mm (*x* axis), 80 mm (*y* axis), and 160 mm (*z* axis), with a resolution of 2560 × 1620 pixels (2K), precision on the XY axes of 0.01–0.05 mm, and layer thicknesses ranging from 0.01 to 0.05 mm.

#### 2.4.6. Processing Samples After Collection

Once printed, the samples need a cleaning procedure to eliminate surplus resin that lingers beneath the surface of the printed item. In order to achieve this, the items were immersed in isopropyl alcohol and cleaned using a soft-bristled brush. Following the cleaning procedure, the components underwent exposure to ultraviolet (UV) light, intended to execute the resin post-curing process. The samples were exposed to UV light (405 nm) for 12 h, and they were rotated every 30 min.

### 2.5. Thermal Analysis

#### 2.5.1. Thermogravimetric Analysis (TGA)

Thermogravimetric analysis was performed to obtain the thermal properties of the composites, photopolymerizable resin, and jute fiber for the different evaluation groups. The test was conducted in a nitrogen atmosphere at a flow rate of 50 mL/min, with a temperature range of 25 °C to 800 °C at a heating rate of 5 °C/min. A TA Instruments model DTG-60H, belonging to the Materials Technology Group of the Navy Research Institute (IPqM), was used. The samples were tested according to the ASTM E1131 [29].

#### 2.5.2. Differential Scanning Calorimetry (DSC)

For DSC analysis, the composites, photopolymerizable resin, and jute fibers were crushed and placed in an aluminum crucible for analysis using a Shimadzu DSC-60A Plus equipment belonging to the Materials Technology Group of the Navy Research Institute (IPqM). A sample containing 3.2 mg of the material of interest was used in a nitrogen atmosphere with a flow rate of 50 mL/min and a heating rate of 5 °C/min in the range of 30 to 550 °C.

#### 2.5.3. Thermomechanical Analysis (TMA)

The determination of the thermal expansion coefficients of the composites and the photopolymerizable resin with solid geometry in the different evaluation groups was carried out by TMA. A Shimadzu equipment, model TMA-60, belonging to the Materials Technology Group of the Navy Research Institute (IPqM), was used. The samples were prepared according to the ASTM E831 [30] standard and placed on a quartz support, and the test was conducted in a nitrogen atmosphere, with a temperature range of 25 °C to 150 °C and a fixed compression load of 10 gf.

#### 2.5.4. Dynamic Mechanical Analysis (DMA)

Dynamic mechanical analysis was used to determine the storage modulus (E’), loss modulus (E”), and tangent delta (Tanδ) parameters, as well as to directly determine the glass transition temperature (T_g_) of the materials studied. The test was developed according to ASTM D4065 [31] standard, using Ta Instruments DMA Q800 equipment, belonging to the Eloísa Mano Institute of Macromolecules (IMA/UFRJ). The samples were tested with dimensions of 30 × 13 × 3 mm with solid and auxetic geometry in the mass fractions of 0, 2%, 2.5%, and 3% of jute fiber. A single cantilever clamp was used, in the temperature range of 0 °C to 160 °C at a heating rate of 5 °C/min in a nitrogen atmosphere, frequency of 1 Hz, and deformation of 0.05%.

### 2.6. Fourier Transform Infrared Spectrometry (FTIR)

Fourier transform infrared spectrometry analysis was used to identify and evaluate the intermolecular vibrations present in the materials under study. FTIR analyses were performed at the Central Laboratory of the Materials Technology Group of the Navy Research Institute (IPqM), using a Thermo Scientific spectrometer with an attached ATR module and OMNIC Spectra^®^ software. Scans were performed in the infrared region between 400 cm^−1^ and 4000 cm^−1^ with a resolution of 4 cm^−1^ and 64 scans in each test.

### 2.7. Scanning Electron Microscopy (SEM)

To verify the good interaction between the fiber and the matrix after the thermal tests, the fractured samples were analyzed by SEM in a Quanta FEG 250 FEI model (Thermo Fisher Scientific, Waltham, MA, USA) operating with secondary electrons at 10 kV.

## 3. Results and Discussion

### 3.1. Thermal Characterizations

The acrylated resin and composites with jute fibers were subjected to thermal tests of TG, DSC, DMA, and TMA, with the aim of gathering information inherent to the thermal behavior of these materials. The analyses related to the evaluated materials will be presented in the following subsections.

#### 3.1.1. Thermogravimetric Analysis (TGA)

**Figure 5:**
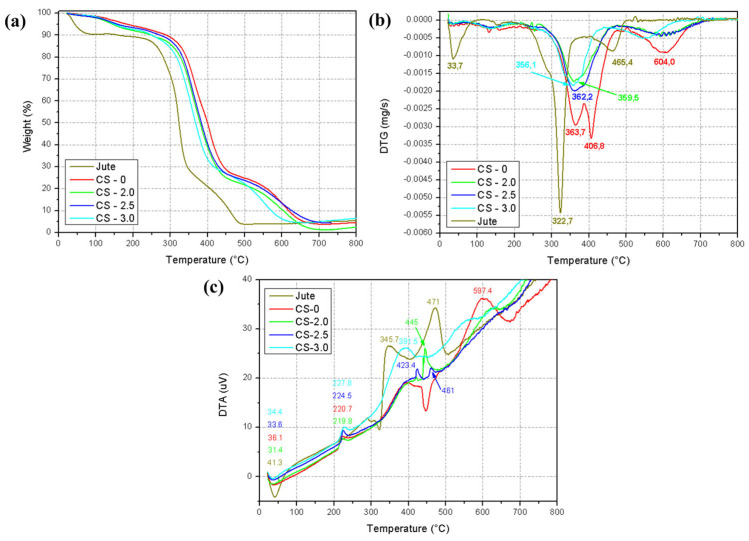
Thermal analysis curves for the evaluated materials: (**a**) TGA, (**b**) DTG, and (**c**) DTA.

Thermogravimetric analysis was performed on jute fibers, resin, and composites in mass fractions of 2%, 2.5%, and 3% of jute fibers. The TGA, DTG, and DTA curves obtained for the respective materials are shown in Figure 5.

The thermal events presented by the TGA curve (Figure 5a) are best interpreted through Table 5.

The composites present better thermal stability at low temperatures (~200 °C) in relation to jute fibers, since the fibers lost 10.75% of their mass, and the composites presented mass loss between 6.6 and 8.1% up to 200 °C. The resin proved to be more stable at this temperature with a mass loss of 5.73%.

The increase in the proportion of jute present in the composites results in a reduction in the degradation onset temperature (326.3 °C) and maximum degradation rate temperature (364.6 °C) of the epoxy resin. These thermal events occur in the ranges of 313.2 to 325.2 °C for the degradation onset temperature and 357 °C to 361.8 °C for the maximum degradation rate temperature. This phenomenon occurs due to the addition of jute fibers to the resin, since jute presents significant degradation at lower temperatures (~300 °C), due to the decomposition of lignocellulosic constituents present in the fibers [32].

It is important to highlight that resin degradation occurs in a higher temperature range (350–450 °C), characterizing the decomposition of the polymer matrix. With the addition of jute (CS-2.0, CS-2.5, CS-3.0), the TGA curve shows a small reduction in the initial thermal stability (around 300 °C), due to the presence of jute components and a more pronounced degradation at intermediate temperatures (~350–400 °C), associated with the interaction between jute and the matrix [33].

By analyzing the DTG curve in Figure 5b, two peaks of interest were observed in the jute fiber curve, the first at 33.7 °C, which indicates the temperature range at which water loss begins in the fibers. The second peak was at 322.7 °C, which indicates the maximum rate of fiber degradation. This temperature is generally associated with the degradation of the lignocellulosic constituents (cellulose and lignin) of natural fibers [34].

For the pure resin (CS-0), a peak is recorded at 363.7 °C, which indicates the maximum rate of polymer degradation in that temperature range, generally associated with the beginning of the mobility of the polymer chains followed by their degradation with increasing temperature.

For the composite curves in general, two distinct peaks were recorded, one at ~359 °C (reduced in comparison to the pure matrix), related to the interaction of jute with the matrix. Another was recorded at ~406 °C, indicating the degradation of more resistant fractions of the composite.

From the DTA curve, it was observed that all materials present an endothermic peak between 30 and 40 °C. This event is associated with the loss of adsorbed water or residual moisture in the materials. Therefore, jute fiber (41.3 °C) presents a more intense peak in this region, due to its hydrophilic character.

In intermediate regions (200–400 °C), the pure resin presents an exothermic peak at 220.7 °C, while the composites with jute show two thermal events: the first exothermic peak (~219–227 °C), indicating partial degradation of the matrix and interaction with the jute. The second exothermic peak (~345–359 °C) is related to the decomposition of lignocellulosic components of the jute. Thus, the presence of jute introduces a new thermal event in the intermediate range (~350 °C), representing the decomposition of fiber components, such as hemicellulose and cellulose.

For higher temperature regions (400–600 °C), the CS-0 curve presents an intense exothermic event at ~597.4 °C, associated with the decomposition of the residual polymer matrix. The composites show additional exothermic peaks, where 423–445 °C highlights the decomposition of lignin or lignocellulosic residues, while 461 to 471 °C indicate the thermal stabilization caused by jute, which slows down the decomposition of the matrix.

In view of the above, it is observed that the overall thermal stability of the matrix is slightly reduced at low temperatures (<350 °C) due to the degradation of the jute components. However, stability at high temperatures (>400 °C) is preserved. The interaction between the photopolymerizable resin and jute is evident by the changes in the DTG and DTA profiles, with multiple degradation peaks. The presence of jute does not significantly compromise the thermal behavior at higher temperatures, indicating that the composite may be suitable for moderately thermal applications.

Going further, thermogravimetric analysis (TGA), complemented by DTG and DTA curves (Figure 5), revealed that all the composites produced presented good thermal stability, with an initial degradation temperature above 310 °C (Table 5). These values indicate that the materials are potentially suitable for engineering applications subject to moderate temperature environments, such as structural components exposed to operational heat in electronic or automotive devices.

It is observed that the raw jute sample presents a distinct thermal degradation behavior, characterized by two main stages. The first occurs at around 283 °C, associated with the decomposition of amorphous components, such as hemicellulose and part of the lignin. The second stage, at around 322 °C, is related to the degradation of cellulose, a more crystalline and thermostable component. This two-phase behavior is typical of lignocellulosic fibers and highlights their heterogeneous nature.

In the reinforced composites (CS-2.0, CS-2.5, and CS-3.0), the DTG curve shows degradation peaks with small variations in relation to the pure resin (CS-0), reflecting the influence of the incorporation of jute on the thermal stability of the system. In particular, sample CS-3.0 presented the lowest degradation temperature (313.2 °C), which can be attributed to the higher proportion of lignocellulosic fraction, whose degradation begins at lower temperatures than the pure polymer matrix. Despite this slight reduction, the reinforced composites maintain excellent thermal performance up to approximately 300 °C, indicating that the addition of fibers does not substantially compromise the thermal integrity of the materials.

Additionally, the total mass loss of the reinforced samples up to 800 °C was lower than that observed for the pure jute sample, reflecting the stabilizing action of the cured acrylate matrix. This result corroborates the good integration between the phases and reinforces the applicability of the composites in scenarios where thermal resistance is required, within moderate operational limits.

#### 3.1.2. Differential Scanning Calorimetry (DSC)

**Figure 6:**
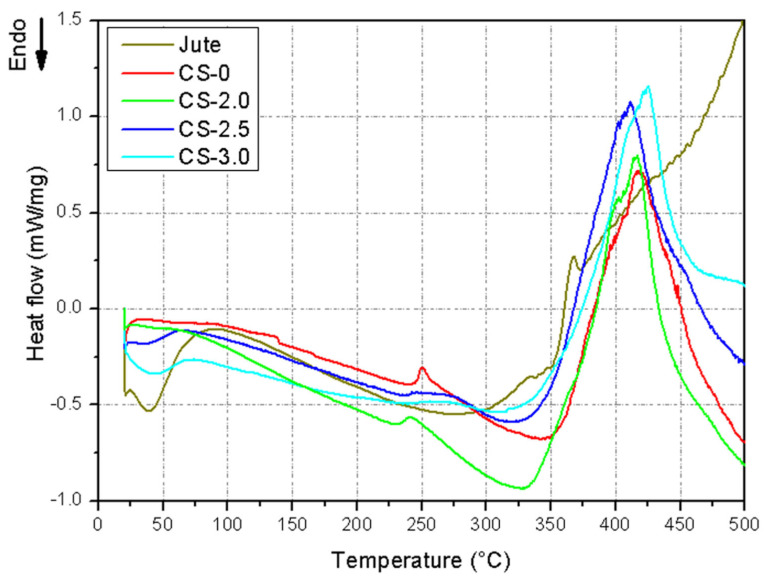
DSC curve for the composites produced in relation to the curves of jute and photopolymerizable resin.

DSC analysis was performed on jute fibers, resin, and composites in mass fractions of 2%, 2.5%, and 3% jute fibers; the curves obtained for the respective materials are shown in Figure 6.

Through the analysis of Figure 6, it was observed that, at low temperatures (<200 °C), the jute fibers present a significant endothermic peak around 50–100 °C, associated with moisture loss. The pure resin (CS-0) shows stable behavior in this region, without significant transitions, while, in the composites (CS-2.0, CS-2.5, and CS-3.0), this endothermic peak also appears but with less intensity compared to pure jute. The presence of jute in the composites contributes to moisture absorption, reflected by an endothermic peak at low temperatures [35].

**Figure 7:**
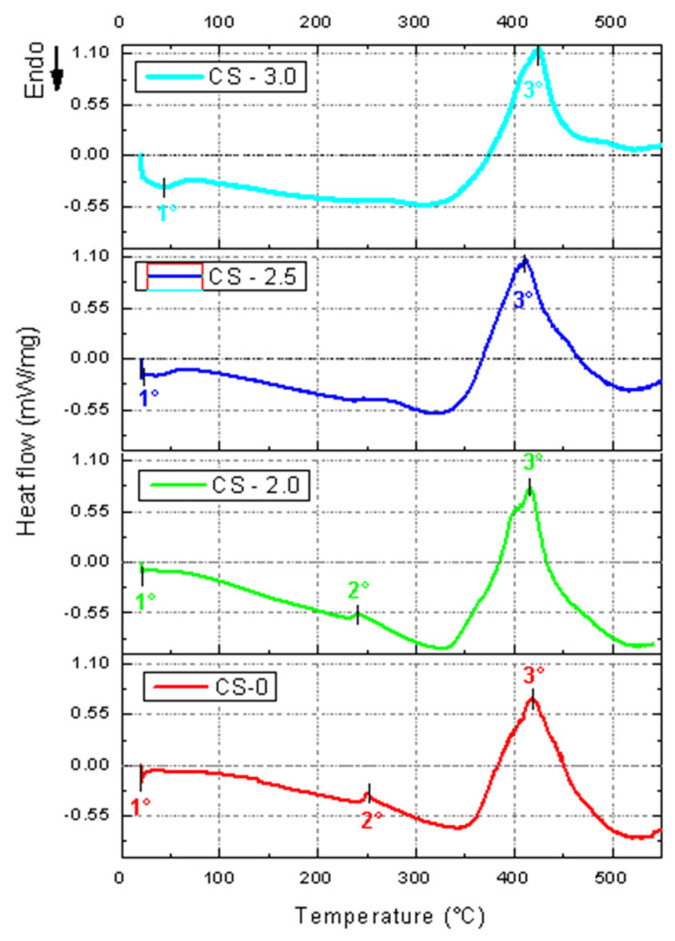
DSC curves of the samples plotted separately.

For better interpretation of the thermal events occurring in the pure resin and in the composites, the curves were plotted in Stack form, as shown in Figure 7 and Table 6.

The first thermal event is related to the loss of adsorbed water or residual moisture in the evaluated samples. In CS-0 (19.7 °C), the peak of lowest intensity occurs due to the absence of natural fibers. In CS-2.0 (30.8 °C), the peak shifts to a higher temperature and reduces its intensity. For CS-2.5 (21.6 °C), it presents the most intense peak (−153.0 mJ), indicating a greater amount of moisture associated with the matrix. CS-3.0 (21.3 °C) reports a reduction in intensity in relation to CS-2.5, but it is still significant (−98.3 mJ). The presence of jute fiber increases moisture retention in the material, being more evident in CS-2.5, possibly due to the greater matrix–fiber interaction at this concentration [35].

In the intermediate thermal event (second event), a partial decomposition of hemicellulose and initial interactions between the matrix and the fiber occur [36]. The second thermal event, identified between approximately 230 °C and 250 °C, should not be interpreted as a degradation process, as erroneously reported in the literature in some cases. In photopolymerizable systems, such as those used in DLP additive manufacturing, events in this temperature range are generally associated with secondary phase transitions, such as segmental relaxations of the polymer matrix, or even with residual curing processes, attributable to the late conversion of remaining acrylate or methacrylate groups. The suppression or significant reduction in this event in the CS-2.5 and CS-3.0 formulations suggests that the presence of jute fibers interferes with the mobility of the matrix, altering the dynamics of molecular rearrangements or limiting the occurrence of remnant curing reactions, possibly due to optical shadowing of the fibers, which reduces the efficiency of polymerization during printing.

The third thermal event (high temperature) is characterized by the recorded exothermic peak, which is related to the decomposition of lignin present in the jute [20]. The third and most evident thermal event, observed in the range of 375 °C to 450 °C, presents an exothermic character and is associated with a set of overlapping phenomena. Firstly, the occurrence of delayed thermal curing stands out, common in partially cross-linked photopolymerizable resins, especially in internal regions of the composites, where the penetration of UV light is limited by the presence of fibers. This effect is intensified in samples CS-2.5 and CS-3.0, which present exothermic peaks of greater intensity. Simultaneously, the beginning of the thermal decomposition of the lignin present in the jute fibers is observed, an exothermic process due to the formation of carbonaceous structures and aromatic condensation reactions. The increasing intensity of this peak in the formulations with higher fiber content indicates that the thermal contribution associated with lignin becomes significant.

In general, the thermal behavior shown in the DSC curves suggests that the incorporation of jute not only alters the hygroscopic capacity of the material but also directly interferes with the efficiency of the photopolymerizable curing and the thermal stability of the system. This behavior highlights the importance of considering adjustments in the curing protocol, especially additional thermal or UV post-processes, in order to ensure the complete conversion of the polymer matrix into systems reinforced with lignocellulosic fibers.

#### 3.1.3. Dynamic Mechanical Analysis (DMA)

**Figure 8:**
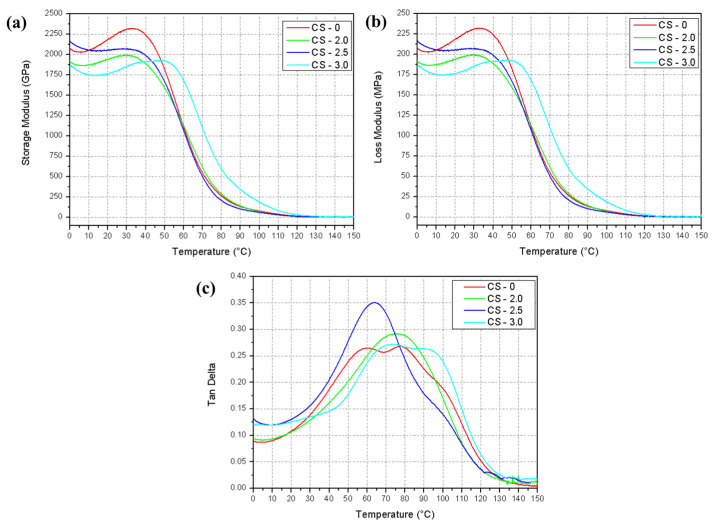
Curves obtained through DMA for solid geometry composite: (**a**) Storage modulus. (**b**) Loss modulus. (**c**) Tan delta.

Dynamic mechanical analyses were performed on the composites with 0%, 2%, 2.5%, and 3% of jute fiber mass for the composites in solid geometry. The storage modulus (E’), loss modulus (E”), and tangent delta (Tan δ) curves were analyzed in each condition. The data obtained are presented in Table 7 and Figure 8.

The storage modulus (E’) reflects the ability of a material to store elastic energy during deformation [34]. The pure resin has the highest initial modulus at 0 °C (~2078.6 MPa), evidencing greater rigidity at low temperatures. For the composite with 2% jute, E’ decreases to ~1911.4 MPa, indicating a reduction in rigidity due to the incorporation of jute at 0 °C. In the CS-2.5 samples (2.5% jute), there is a partial recovery of rigidity, with E’ at 0 °C rising to ~2163.4 MPa, indicating a good matrix–fiber interaction at this concentration. The sample with 3% jute has the lowest initial modulus (0 °C) (~1836.7 MPa), suggesting that higher concentrations of jute may compromise the uniformity of the matrix [37].

The loss modulus is related to the energy dissipated in molecular movements with the increase in temperature experienced by the material, indicating the material’s damping capacity [38]. Sample CS-0 presented higher E” at 32.8 °C (~232.0 MPa), indicating greater energy dissipation in the pure matrix. Composites CS-2.0 (31.2 °C) and CS-3.0 (46.9 °C) presented reduced values (~199.1 MPa and ~192.9 MPa, respectively), suggesting lower dissipation capacity with the presence of jute. However, sample CS-2.5 presents a partial recovery of E” (~207.3 MPa), demonstrating a good balance and interaction between the matrix and the fiber.

Thus, the incorporation of jute reduces the energy dissipation capacity (E”), but, at intermediate fiber concentrations (CS-2.5), the system maintains adequate damping characteristics. The tangent delta (Tan δ) is the ratio between E” and E’, indicating the damping efficiency and the glass transition (T_g_) of the material [39]. Some of the tangent delta curves recorded in Figure 8c present two Tan δ peaks for the same material; this phenomenon characterizes a second T_g_, or post-curing temperature of the material [34]. Thus, Tg1 (first peak) for sample CS-0 is 59.8 °C, and, for sample CS-3.0, Tg1 increases significantly to 72.8 °C, indicating greater thermal rigidity due to the presence of jute. For the second peak recording Tg2, for CS-0, Tg2 = 77.6 °C is obtained, and, for CS-3.0, Tg2 increases to 90.1 °C, evidencing greater thermal stability of the composite. Regarding the height of the Tan δ peaks, sample CS-2.5 presents the highest Tan δ peak (~0.35), indicating the best damping capacity. Thus, the addition of jute increases the glass transition temperature (T_g_), especially in CS-3.0, reflecting greater thermal stability. CS-2.5 presents the best damping performance, suggesting an optimized matrix–fiber interaction.

The observation that the damping performance (tan δ) was optimized at 2.5% fibers (CS-2.5) suggests, indeed, a more efficient interaction between the photocurable polymer matrix and the jute fibers at this ratio. This condition appears to offer an ideal balance between the stiffness imposed by the fibers and the mobility of the matrix, resulting in greater mechanical energy dissipation. The more pronounced tan δ peak observed in Figure 8c indicates a more efficient molecular relaxation and, therefore, a better damping capacity.

On the other hand, the composite with 3% fibers (CS-3.0), although presenting the highest secondary glass transition temperature value (Tg2 = 90.1 °C), has lower tan δ values, which can be interpreted as a reduction in the capacity to dissipate energy through internal friction. This reduction may be associated with a lower segmental mobility of the matrix, possibly caused by poor fiber dispersion or the formation of agglomerates. These agglomerates act as stress concentration zones and delamination points, impairing the overall performance of the composite and indicating a less effective matrix–reinforcement interaction.

Furthermore, the significant increase in the coefficient of thermal expansion (α = 46.9 °C) in CS-3.0 reinforces the hypothesis of lower interfacial compatibility, since high values of α suggest marked dimensional instability, resulting from the lower capacity of the matrix to restrict the thermal movements of the incorporated fibers.

Therefore, the comparison between the samples suggests that the condition with 3% fibers presents a less efficient interaction between the matrix and the reinforcement, compared to the proportion of 2.5%, the latter being the one that best reconciled stiffness, molecular mobility and dynamic damping. This highlights the importance of optimizing the fiber content to maximize the functional performance of lignocellulosic composites in photocurable polymer systems.

Going further, the incorporation of natural fibers into photopolymerizable resins, such as those used in the Digital Light Processing (DLP) process, presents relevant technical challenges, especially with regard to the dispersibility and uniformity of the reinforcement within the polymer matrix. Homogeneous fiber distribution is essential to ensure the reproducibility of the mechanical and thermal properties of the composite, since agglomerates or zones of high fiber concentration can act as failure initiators, compromising the structural integrity of the material. In this context, the mass fraction of jute fibers was limited to 3% in the present study, based on technical and operational factors observed during sample preparation and printing.

One of the main limitations is the increase in resin viscosity with the incorporation of higher fiber contents, especially when these are short and untreated. This increase in viscosity compromises the fluidity required for uniform layer deposition during the DLP process, which can cause interlaminar adhesion failures and internal defects in the printed composite [40]. Another limiting factor refers to the sedimentation and agglomeration of fibers. When in high concentration and without surface treatment, fibers tend to sediment or form agglomerates in the resin before curing, which impairs homogeneity and, consequently, the mechanical properties of the final material [41].

Additionally, the increased fiber load negatively impacts the optical transmittance of the resin, hindering the penetration of ultraviolet light during photopolymerization. This can result in incomplete or partial curing of the matrix, leading to undercured regions with low densification and reduced dimensional stability [42]. The presence of fibers in higher contents can also compromise the interfacial adhesion between reinforcement and matrix, increasing the susceptibility to delamination and premature failure under loading, especially if the interface is not previously modified by chemical treatments or compatibilizers.

#### 3.1.4. Thermomechanical Analysis (TMA)

**Figure 9:**
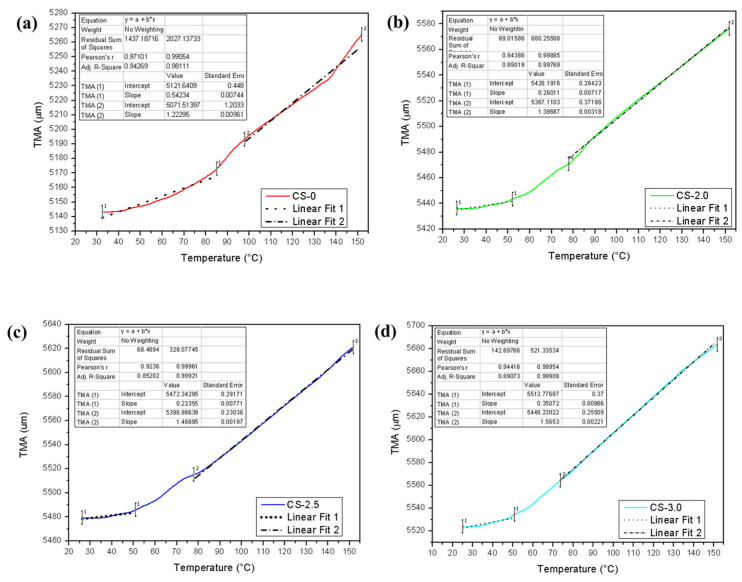
TMA curves and thermal expansion coefficient of photopolymerizable resin and solid geometry composites. (**a**) CS-0. (**b**) CS-2.0. (**c**) CS-2.5. (**d**) CS-3.0.

The samples in solid geometry were subjected to thermomechanical analysis, and the results obtained are presented in Table 8 and Figure 9.

**Figure 10:**
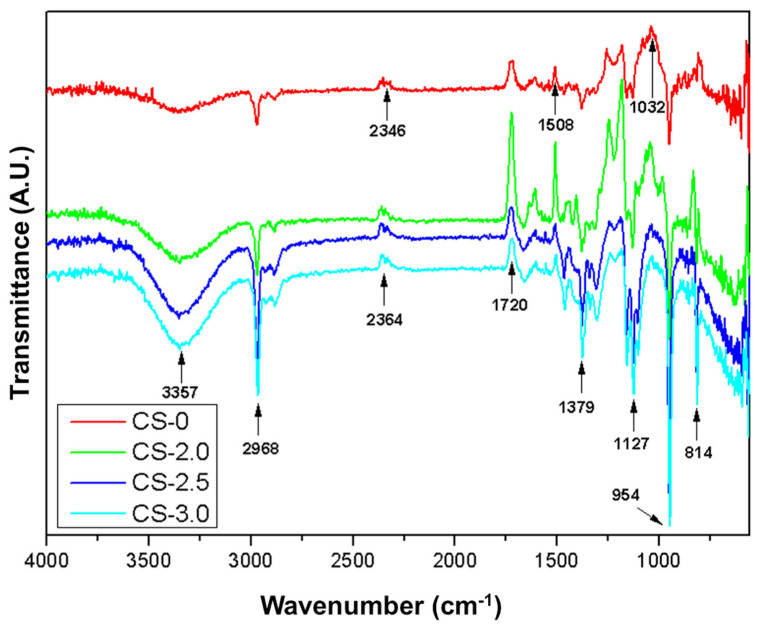
FTIR spectra generated for photopolymerizable resin and composites with jute fibers.

The CTE represents the material’s ability to expand with increasing temperature; thus, high values indicate greater thermal expansion [26]. Through the analysis of Table 8 and Figure 10, it was possible to identify the thermal expansion behavior of the composite samples in solid geometry before (CTE1) and after (CTE2) the glass transition temperatures 1 and 2 observed in the materials. Thus, among the evaluated samples, the pure resin presents high thermal stability at the beginning of the expansion (0.105 µm/°C), since it was the highest CTE1 value obtained among all the samples in this temperature range. The samples with 2% jute presented a CTE1 of 0.047 µm/°C, highlighting the drastic reduction in the CTE due to the presence of jute in relation to the polymer matrix. In the CS-2.5 condition, a CTE1 of 0.040 µm/°C was observed. The CS-3.0 composite presents a CTE1 = 0.063 µm/°C, highlighting an increase in the thermal expansion in relation to CS-2.5 but still lower than CS-0.

For the CTE2 region, the pure resin presented a value of 0.239 µm/°C, highlighting a marked thermal expansion at high temperatures. The composite with 2% jute CS-2.0 presented a moderate increase in relation to the pure resin, presenting a CTE2 of 0.255 µm/°C. For the CS-2.5 samples, the thermal expansion continued to increase, with a CTE2 of 0.267 µm/°C. The CS-3.0 condition presented maximum thermal expansion in the final interval, with a CTE2 value of 0.283 µm/°C.

Regarding the glass transition temperatures in the first stage, they were between 57.6 °C (CS-2.0) and 58.8 °C (CS-0). This transformation temperature is generally associated with the point where the material passes from a glassy state to a more elastic state [43]. The sample with 2.5% jute presents Tg1 of 58.7 °C, highlighting the recovery of thermal stability in relation to sample CS-2.0. A new reduction in Tg1 is observed for the CS-3.0 composite, since Tg1 is 57.3 °C. This reduction may be associated with the increase in heterogeneity in the composite caused by the increase in the fraction of fibers present [43].

At Tg2, the pure resin presents high thermal stability among the evaluated samples, with Tg2 of 93.1 °C. The composites with 2%, 2.5%, and 3.0% presented a reduction in Tg2, with respective values of 74.6 °C, 73.2 °C, and 67.8 °C. This indicates that the presence of jute resulted in a reduction in the glass transition temperature in the second stage, providing its occurrence at lower temperatures.

It is important to highlight that all linear adjustments used to determine the thermal expansion coefficients in the respective curves presented values close to 1 (0.923~0.999), indicating the linearity of the curves in the calculated intervals.

It is clear that the incorporation of lignocellulosic fibers, such as jute, into photopolymerizable matrices produced by Digital Light Processing (DLP) is often highlighted in the literature as a promising strategy for the production of sustainable materials. However, a critical analysis of the results obtained allows us to observe that the simple addition of small fractions of natural fibers, by itself, is not enough to classify these composites as environmentally sustainable in the broadest sense.

From the point of view of thermal properties, the TGA results demonstrate that the introduction of fibers promotes a reduction in the initial degradation temperature, which varies from 326.3 °C for the pure matrix (CS-0) to 313.2 °C in the composite with 3% fibers (CS-3.0). This reduction is directly associated with the chemical nature of the lignocellulosic fibers, especially the presence of hemicellulose, amorphous cellulose, and lignin, which have lower thermal stability compared to the photopolymerizable matrix. Similarly, the DMA results indicate a slight decrease in the storage modulus (E’), which reduces from 2.31 GPa to 1.92 GPa, reflecting a slight loss of structural rigidity associated with the introduction of fibers.

This observation highlights an important issue: the addition of jute fibers, when carried out in low volume fractions (up to 3%), does not have as its main function structural reinforcement in the classical sense but rather the introduction of specific attributes, such as dimensional stability (TMA), lightness, and reduced cost. However, such benefits are not sufficient to characterize, in isolation, a substantial advance in terms of environmental sustainability.

The sustainability of a polymer composite does not depend exclusively on the type of reinforcement used but is intrinsically related to the nature of the matrix, the possibility of recycling or biodegradation at the end of the material’s useful life, and the impact of its complete life cycle. In the case of conventional photopolymerizable matrices used in DLP, a significant limitation is observed, since they are thermosetting materials, derived mainly from petrochemical sources, non-recyclable after curing, and have low biodegradation capacity.

Therefore, the contribution of jute fibers to the sustainability of the system, although positive, is marginal when considered in isolation. The use of 2–3% natural fibers does not substantially alter the non-biodegradable nature of the matrix, nor does it modify its dependence on fossil resources. In order to apply the concept of sustainability robustly, one of the most promising approaches involves replacing the conventional matrix with photopolymers derived from renewable sources, using monomers obtained from biomass, such as lignin, vanillin, and vegetable oils. These biobased systems not only reduce dependence on fossil resources but also enable the creation of materials with a lower carbon footprint and, potentially, greater biodegradability. In parallel, the incorporation of vitrimer-based technologies represents a significant advance, allowing materials traditionally classified as thermosets to become reprocessable and recyclable. This is due to the introduction of polymer networks endowed with dynamic covalent chemistry, capable of reorganizing their bonds under external stimuli, such as heat or catalysts, without a significant loss of performance.

Furthermore, it is essential to increase the volumetric fraction of fibers, associated with the implementation of techniques that ensure homogeneous dispersion and efficient interfacial compatibility. This not only contributes to improving mechanical and thermal properties but also substantially reduces the content of synthetic polymers in the final formulation, expanding the environmental benefits of the composite. Finally, the adoption of robust Life Cycle Assessment (LCA) tools is essential for the accurate quantification of environmental impacts throughout all stages of the material’s life cycle, from obtaining raw materials to their disposal or recycling. This approach is essential to validate, in a scientific and transparent manner, the effectiveness of the proposed solutions in terms of sustainability and the responsible development of engineering materials.

### 3.2. FTIR Analysis

The composites and the photopolymerizable resin were subjected to FTIR analysis; the spectra obtained are shown in Figure 10.

In Figure 10, it is possible to compare the spectra of the photopolymerizable resin and the respective composites with jute fibers, by identifying the absorption bands relative to the constituents of the materials analyzed. When observing the spectrum in question, a sharp band is noted at 3357 cm^−1^. This vibration can be attributed to the O-H (alcohol or carboxylic acid) or N-H (amines) stretching vibration, which is common in resins containing reactive components or modifiers [44]. In the region of 2968 cm^−1^, the C-H stretching generally occurs, characteristic of methyl and methylene groups in acrylic or methacrylic resins. The band recorded at 1720 cm^−1^ can be associated with the C=O stretching, indicating the presence of carbonyls (esters or acrylates) in photopolymerizable resins. This is an important peak for acrylic resins, as in the case of the resin under study [45]. Deformation vibrations associated with aromatic groups or C-H stretching in methyl systems are generally associated with bands in the region of 1508 and 1379 cm^−1^. In the region of 1260–1032 cm^−1^, C-O stretching (esters, ethers, or alcohols) is recorded. This region confirms the presence of ester or acrylic bonds in the resin structure [46]. For vibrations at 814 cm^−1^, it can be attributed to out-of-plane deformations of =C-H bonds (unsaturated carbons, as in acrylates).

**Figure 11:**
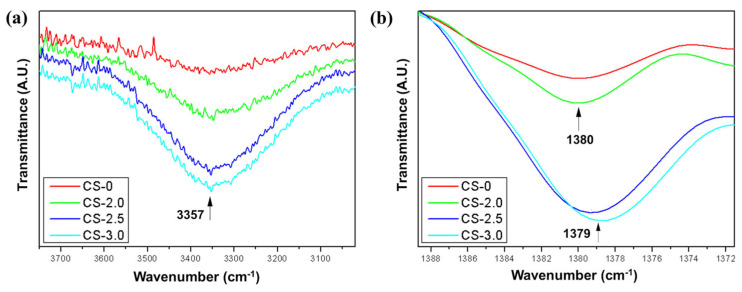
Absorption band at 3357 cm^−1^ (**a**,**b**), absorption bands at 1379 to 1380 cm^−1^ for the evaluated samples.

For a better understanding of the influence of the addition of jute fibers in the photopolymerizable resin, a cutout was made in two regions of interest in the spectrum of Figure 10; these cutouts are shown in Figure 11.

In the region of 3357 cm^−1^, the band is attributed to the stretching of O-H groups (hydroxyls), presenting an increase in transmittance as the jute concentration increases. The CS-0 curve (pure resin) has the lowest transmittance in this band, while CS-3.0 (3% jute) presents the highest transmittance. The increase in transmittance suggests interactions between the hydroxyl groups of the resin and the jute fibers, indicating possible formation of hydrogen bonds or reactions between the matrix and the chemical groups present in the jute [44,47].

In the region of 1379–1380 cm^−1^, C-H deformation occurs in methyl groups, where small changes in the transmittance of the bands were observed. In the CS-0 curve, the band presents a slight shift to 1380 cm^−1^, while, in CS-2.0, CS-2.5, and CS-3.0, the band shifts slightly to 1379 cm^−1^, and its transmittance increases progressively. The increase in transmittance and the slight shift in the band may be associated with interactions between the C-H groups of the resin and the jute, as well as possible restrictions in the movement of the polymer chains due to the incorporation of the fibers, making the material more rigid.

In general, jute fibers provide a variation in the transmittance of specific bands of the acrylated photopolymerizable resin, suggesting a change in the chemical and structural properties of the resin. Thus, the insertion of jute fibers appears to reduce the contribution of free polymeric groups and creates a more rigid and interconnected structure, compatible with the expected behavior for reinforced composites.

### 3.3. SEM Analysis

**Figure 12:**
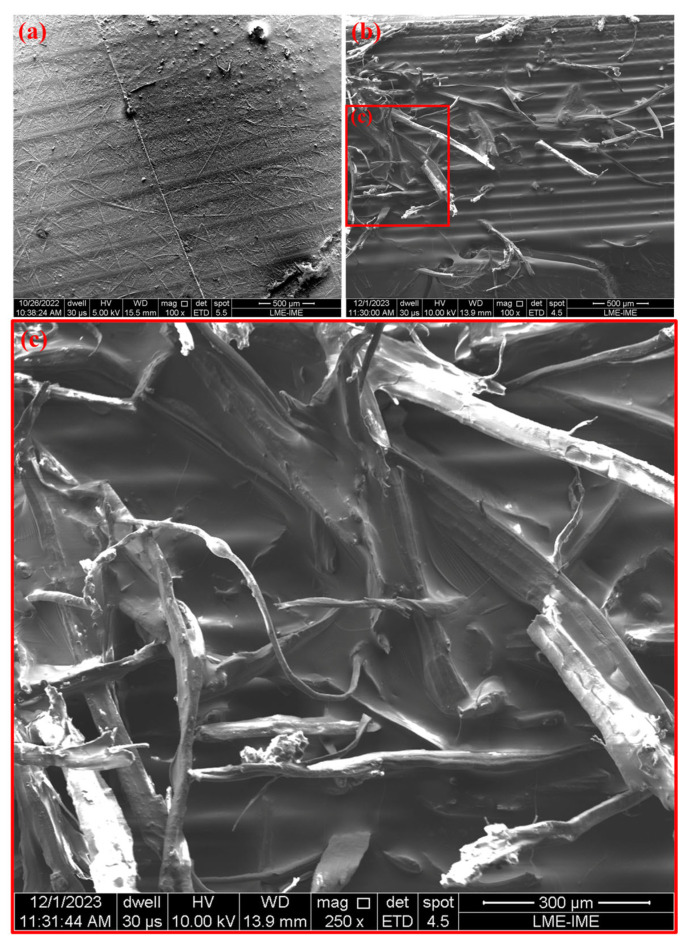
SEM of the fracture surfaces of the tested specimens: CS-0 (**a**) 100×; CS-2.5 (**b**) 100×; and (**c**) 250×.

The micrographs obtained by Scanning Electron Microscopy (SEM) in Figure 12 show the fracture surfaces of the composites and the pure resin, making it possible to extract important information about the interfacial interaction between the photopolymerizable matrix and the jute fibers.

In Figure 12a, corresponding to the pure resin, a relatively smooth surface is observed, with few defects and typical characteristics of brittle fracture. This morphology is consistent with the nature of cross-linked photopolymers, which present a high degree of rigidity and low plastic deformation capacity, which corroborates the DMA and TGA data obtained. In Figure 12b, which represents the composite with 2.5% jute fibers, a network of fibers dispersed in the matrix can be clearly seen, where some are partially embedded and others present regions where the matrix adheres to the fiber surfaces. In the detail with greater magnification in Figure 12c, it is possible to observe signs of physical anchoring of the matrix on the fibers, as well as regions where the extraction of the fibers left cavities, indicating that the failure occurred, in part, by interfacial mechanisms.

The observed interaction is predominantly physical, based on mechanical anchoring promoted by the surface roughness of the jute fiber and by the filling of the matrix in the alveoli and irregularities of the fibers. The nature of jute, with high porosity and rough topography, favors this type of interaction. However, the occurrence of secondary chemical interactions, especially of the hydrogen bond type, between the hydroxyl groups (–OH) on the surface of the lignocellulosic fibers and the polar groups present in the acrylated matrix after photopolymerization cannot be ruled out. However, since there was no indication of prior chemical functionalization of the fibers (such as silanization or alkaline treatments), it is unlikely that there was a formation of stable covalent bonds between the fiber and matrix in the system analyzed.

## 4. Conclusions

Based on the results obtained, it is concluded that the incorporation of jute fibers in photopolymerizable composites produced via DLP significantly influences the thermal, mechanical, and chemical properties of the material. The addition of up to 3% by the weight of fibers resulted in a slight reduction in thermal stability and storage modulus while promoting greater dimensional stability and demonstrating chemical interactions between the constituents, as demonstrated by FTIR and DMA analyses. These findings indicate that small fractions of natural fibers can be efficiently integrated into UV-curable systems without substantially compromising the integrity of the composite, enabling their application in moderate temperature environments.

For future applications, these sustainable composites can be used in lightweight structural components for sectors such as automation, electronics, consumer goods, and architecture, where moderate thermal resistance and low environmental impact are desirable. Future studies may expand the scope of research by exploring fiber surface treatments, low-viscosity resin formulations, and new dispersion techniques, aiming to increase the reinforcement fraction without compromising processability or final properties, thus expanding the viability of lignocellulosic composites in high-resolution additive manufacturing technologies.

## Figures and Tables

**Table 1:** Details regarding the technical specifications of the 3D fila light-curing resin.

General Characteristics		
Product name	–	Substance FS acrylated resin
Appearance	–	Blue liquid
Acrylated monomers	–	>90%
Photo initiators	–	<5%
Pigmentation	–	<2%
Odor	–	Smooth, like an ester
Color	–	Blue
pH	–	6.8–7.3
Melting point	–	<0 °C
Boiling point	–	>200 °C
Flash point	–	150 °C
Vapor pressure	–	<0.01 kPa at 25 °C
Relative density	–	1.08–1.14 g/cm^3^
Viscosity	–	190–500 cps at 25 °C
Specific mass at 20 °C	–	1122.2 kg/m^3^
Light band/polymerization	–	405 nm

**Table 2:** Ratio of mass and volume of photopolymerizable resin concerning jute mass for producing composites.

Mass Fraction of Jute Fibers (%)	Volume	Mass		
	Resin (mL)	Resin (g)	Fibers (g)	Total (g)
0	125	192.8	0	192.8
2.0	122.5	188.94	3.85	192.8
2.5	121.87	187.98	4.82	192.8
3.0	121.25	187.01	5.78	192.8

**Table 3:** Suggested naming convention for the generated samples.

Mass Fraction of Jute Fibers (%)	Solid Geometry
0	CS-0
2.0	CS-2.0
2.5	CS-2.5
3.0	CS-3.0

**Table 4:** Parameters utilized for sample printing.

Parameters	Resin	Composites
Operating temperature	25 °C	25 °C
Bottom exposure time	55 s	60 s
Light off delay	2 s	5 s
Exposure time	3.5 s	5 s
Bottom lifting distance	9 mm	6 mm
Motor speed	1 mm/s	4 mm/s

**Table 5:** Thermogravimetric parameters observed for the evaluated samples.

Samples	A (%)	B (°C)	C (°C)	D (%)	E (%)	F (%)
Jute	10.75	283.0	322.4	79.0	95.9	96.2
CS-0	5.73	326.3	364.6	48.58	86.42	95.5
CS-2.0	8.1	322.4	360.4	61.5	90.02	97.5
CS-2.5	6.6	325.2	361.8	59.5	86.2	95.1
CS-3.0	7.5	313.2	357	66.6	93.6	93.8
^A^ Mass loss up to 200 °C (%)			^E^ Mass loss up to 600 °C (%)			
^B^ T_onset_ of degradation (°C)			^F^ Mass loss up to 800 °C (%)			
^C^ T_Max_ degradation rate (°C)						
^D^ Mass loss up to 400 °C (%)						

**Table 6:** Thermal events observed in DSC for the evaluated samples.

	Thermal Event								
Samples	1°			2°			3°		
	A	B	C	A	B	C	A	B	C
	(°C)	(°C)	(mJ)	(°C)	(°C)	(mJ)	(°C)	(°C)	(J)
CS-0	19.7	19.8	−30.2	244.8	250.4	37.8	416.6	437.1	3.09
CS-2.0	30.8	30.9	−18.5	230.2	243.4	23.4	378.7	427.4	2.18
CS-2.5	21.6	21.9	−153.0	-	-	-	375.0	412.9	2.97
CS-3.0	21.3	42.9	−98.3	-	-	-	375.7	423.9	3.52
^A^ T_onset_			^B^ Peak			^C^ Heat			

**Table 7:** Parameters obtained by DMA observed for samples in solid geometry.

Samples	E’_0_	E’	E_0_”	E”	α	Tan δ	
	(MPa)	(MPa)	(MPa)	(MPa)	(°C)	T_g1_ (°C)	T_g2_ (°C)
CS-0	2078.6	2318.7	207.9	232.0	32.8	59.8	77.6
CS-2.0	1911.4	1887.5	191.1	199.1	31.2	-	75.4
CS-2.5	2163.4	2073.1	216.5	207.3	30.1	63.8	-
CS-3.0	1836.7	1929.2	183.9	192.9	46.9	72.8	90.1

**Table 8:** TMA data obtained for samples in solid geometry.

Samples	CTE_1_	R^2^	CTE_2_	R^2^	TG_1_	TG_2_
	(µm/°C)		(µm/°C)		°C	°C
CS-0	0.105	0.971	0.239	0.990	58.8	93.1
CS-2.0	0.047	0.943	0.255	0.998	57.6	74.6
CS-2.5	0.040	0.923	0.267	0.999	58.7	73.2
CS-3.0	0.063	0.944	0.283	0.999	57.3	67.8

## Data Availability

The original contributions presented in this study are included in this article; further inquiries can be directed to the corresponding author.
